# Stress hormones, social associations and song learning in zebra finches

**DOI:** 10.1098/rstb.2017.0290

**Published:** 2018-08-13

**Authors:** Neeltje J. Boogert, Robert F. Lachlan, Karen A. Spencer, Christopher N. Templeton, Damien R. Farine

**Affiliations:** 1 Centre for Ecology and Conservation, University of Exeter, Penryn Campus, Penryn TR10 9FE, UK; 2 School of Biological and Chemical Sciences, Queen Mary University of London, London E1 4NS, UK; 3 School of Psychology and Neuroscience, University of St. Andrews, St Andrews KY16 9JP, UK; 4 Department of Biology, Pacific University, Forest Grove, OR 97116, USA; 5 Department of Collective Behaviour, Max Planck Institute for Ornithology, Radolfzell 78315, Germany; 6 Chair of Biodiversity and Collective Behaviour, Department of Biology, University of Konstanz, Konstanz 78464, Germany

**Keywords:** developmental stress, information use, social networks, social learning, song learning, stress hormones

## Abstract

The use of information provided by others is a common short-cut adopted to inform decision-making. However, instead of indiscriminately copying others, animals are often selective in what, when and whom they copy. How do they decide which ‘social learning strategy’ to use? Previous research indicates that stress hormone exposure in early life may be important: while juvenile zebra finches copied their parents' behaviour when solving novel foraging tasks, those exposed to elevated levels of corticosterone (CORT) during development copied only unrelated adults. Here, we tested whether this switch in social learning strategy generalizes to vocal learning. In zebra finches, juvenile males often copy their father's song; would CORT-treated juveniles in free-flying aviaries switch to copying songs of other males? We found that CORT-treated juveniles copied their father's song less accurately as compared to control juveniles. We hypothesized that this could be due to having weaker social foraging associations with their fathers, and found that sons that spent less time foraging with their fathers produced less similar songs. Our findings are in line with a novel hypothesis linking early-life stress and social learning: early-life CORT exposure may affect social learning indirectly as a result of the way it shapes social affiliations.

This article is part of the theme issue ‘Causes and consequences of individual differences in cognitive abilities’.

## 1. Introduction

While most studies of learning and cognition are conducted on subjects in social isolation, most wild animals live in a social context—be it a territorial or a gregarious one. Animals use information generated by the behaviour of others in species ranging from fruit flies (*Drosophila melanogaster*) [1] to humans [2]. Social information can shape individuals' decisions when tackling virtually every life challenge, from learning to exploit novel food sources [3] and choosing mates [4], to avoiding brood parasites [5] and predators [6]. However, theory suggests that indiscriminate social information use is not adaptive [7], and accumulating evidence shows that animals employ ‘social learning strategies’ in choosing what, when and whom to copy [8,9]. Yet individuals vary in whether they (appear to) use social information [10] and, if so, in which social learning strategy they adopt [11]. Relatively few studies have investigated the mechanisms underlying this interindividual variation in social information use, but there is accumulating evidence to suggest that early-life conditions [12,13] and social interactions [11,14–18] may be important.

In two recent studies, we investigated how early-life conditions shape social associations and social learning strategies in the highly gregarious zebra finch (*Taeniopygia guttata*). First, we found that juvenile zebra finches that were fed the avian stress hormone corticosterone (CORT) during the nestling phase later formed less exclusive (or more random) social bonds in a colony setting (free-flying aviaries containing six to seven families) relative to their control-treated siblings. In particular, CORT-treated juveniles spent less time foraging with their parents [19]. When presented with a novel foraging task, we next found that while control juveniles tended to copy their parents' behaviour to solve the task, their CORT-treated siblings exclusively copied unrelated adults [20]. This could be because the CORT-treated juveniles may not have perceived their parents as desirable role models, or because the parents may have treated their experimentally stressed (and thus ‘lower quality’) offspring differently. Regardless of the underlying mechanisms, early-life CORT exposure appeared to induce a switch in with whom juveniles affiliated [19] and whom they copied when socially acquiring novel foraging behaviours [20]. The aim of the current study was to determine whether early-life CORT exposure had a similar effect on the social learning of song. If so, we wanted to identify whether this was due to CORT-associated changes in the choice of whom to copy, or a by-product of changes in social affiliation patterns. Bird song is the quintessential example of a socially transmitted trait, and song learning is likely to be important for individuals' fitness: which song an individual sings and how accurately they learn it can have long-lasting consequences for their later ability to compete in territorial disputes or court potential mates [21–23], and can predict individuals’ reproductive success and longevity [24].

In addition to potentially affecting social affiliations and/or song model choices, early-life stress may also influence song learning by impacting cognitive ability. Song learning involves a variety of cognitive processes. Juveniles typically acquire information about species-specific song by listening to the songs of adults (tutors) during a relatively short period (the ‘sensitive window/phase’) in development. They then memorize this information, often for many months, and use it to shape and practise their own song as they mature [25]. Studies that subjected juvenile songbirds to a variety of early-life stressors (e.g. increased brood size, food restriction and CORT administration; reviewed in [26]) often found that stressed males sing lower-quality songs; their songs tend to be shorter, contain fewer (unique) songs or syllables, less accurately copied syllables or syntax, and are perceived as less attractive by females. Some developmental stress studies even report a reduction in the volume of the song control nuclei in the brain [26] (see Pike *et al.* [27] for another example of environmentally induced changes in brain morphology). The ‘developmental stress hypothesis' proposes a potential explanation for these findings [26,28]: song control nucleus development in the brain requires considerable energetic resources during a period of rapid physical and neuronal growth. If these energetic resources are constrained by developmental stressors such as sibling competition, food scarcity or predation threat, then song development is likely to be negatively affected. However, the juvenile males in these developmental stress studies, as in most captive studies on song learning, tend to be experimentally constrained to learn from a single adult tutor. It thus remains to be established whether developmentally stressed males show impoverished song learning in more naturalistic social contexts, such as in colonies where young birds are free to choose to associate with and learn from multiple potential song tutors. The importance of bi-directional interactions between the social environment and cognitive performance is now becoming more widely appreciated [29], and evidence for a critical influence of social context on cognitive performance is accumulating in species ranging from pond snails (*Lymnaea stagnalis*) [30] to Australian magpies (*Cracticus tibicen dorsalis*) [31].

Zebra finches are the foremost model system for studies of song development [32–34]. Male zebra finch song structure and performance play a crucial role in female mate choice in captivity [35] and predict reproductive success in the wild [36]. Males repeatedly sing a single stereotyped and unique song motif during courtship. Captive studies suggest that juvenile males tend to learn these courtship songs from their fathers, if the latter are available as tutors during the sensitive phase when song templates are acquired, i.e. between approximately 35–65 days post-hatching, after they have fledged [37–40]. Zebra finches are also highly gregarious, non-territorial birds that breed in colonies ranging in size from approximately 4 to 136 pairs [41], making them ideal for studying song learning strategies in a dynamic social context. Even so, most experimental studies on zebra finches in captivity have been based on constrained song tutor choice: birds were usually confined to small cages and only given the choice to copy the song of their father or one alternative tutor, without the opportunity to freely associate in a broader social group. The two studies in which breeding pairs and their offspring were kept in free-flying aviaries containing multiple potential tutors [42,43] generated complementary but contradictory findings: Williams [42] found that the majority of juveniles produced songs that did not resemble their father's, and they instead appeared to copy the unrelated males that they interacted with the most. Similarly, Mann & Slater [43] found that most juveniles learnt their songs from the male with whom they maintained greatest proximity, but in contrast to Williams [42], this was often the father. These studies suggest that there could be considerable variation in the choice of song tutor under (semi-) natural rearing conditions, which is likely related to the social associations that young birds experience, and thus their social preferences. Here, we take advantage of being able to quantify fine-scale social associations among all individuals in replicated colonies of zebra finches, combined with experimental manipulations of early-life conditions, to uncover some of the mechanisms that may underlie the observed variation in song tutor selection.

In this study, we examined the relationships between early-life exposure to CORT, fathers' and sons’ social associations, and sons' song tutor choice and song copying accuracy. We used data from the same zebra finches and experimental design as in our previous studies [19,20]: half of the offspring in each of 13 zebra finch families were exposed to experimentally elevated levels of CORT in the nest. After fledging, all individuals’ feeder visits in two aviaries were recorded using an automated tracking system, generating a social foraging network of birds' co-occurrences at the feeders. Next, we generated a ‘song similarity matrix’ between all males in both aviaries. We then combined these data to test three, not necessarily mutually exclusive, predictions drawn from previous studies: (i) in contrast to control juveniles, CORT-treated juveniles will avoid copying their father's song (the ‘tutor choice hypothesis', based on Farine *et al.* [20]); (ii) the more fathers and sons associate during the sensitive phase for song learning, the more similar the sons' songs will be to those of their fathers (the ‘social preference hypothesis', based on Williams [42] and Mann & Slater [43]); and (iii) CORT-treated juveniles will not be capable of copying their father's song as accurately as control juveniles (the ‘cognitive impairment hypothesis', based on Peters *et al.* [26]).

## 2. Material and methods

### (a) Breeding protocol and corticosterone treatment

As described in [19], we housed 24 domesticated adult zebra finch pairs in breeding cages and of these, 13 pairs produced fertile eggs. To facilitate chick age-standardized hormone treatment, we synchronized the within-brood hatching dates by replacing eggs with plastic dummies until the brood was complete. Half of the chicks in each brood were assigned to the CORT treatment following [44]: between days 12 and 28 post-hatching, they were pipette-fed 20 µl of CORT (Sigma-Aldrich; 0.155 mg ml^−1^ in peanut oil) twice daily, giving a total dose of 6.2 µg CORT per day. This dose is known to result in plasma CORT levels comparable to those naturally induced in untreated zebra finch chicks exposed to an acute stressor [44]. Control chicks were fed 20 µl of pure peanut oil when their siblings received CORT. For additional details, see the electronic supplementary material.

### (b) Social networks in aviaries

When chicks were on average ± s.d. = 35 ± 1 days old (range: 33–38 days), we fitted them and their parents with passive integrated transponder (PIT) tags (Dorset ID) attached to unique colour rings and released families together into one of two identical indoor aviaries (3.0 × 3.1 × 3.2 m) on the same day. The aviaries were visually and acoustically isolated from each other. Each aviary contained seven (*N* = 34 birds: 16 females, 18 males) and six families (*N* = 29 birds: 14 females, 15 males), respectively, and both aviaries were equipped with two identical transparent feeders (28 × 28 × 10 cm) containing ad libitum finch seed at all times, except during a 3-day novel foraging task experiment (described in [20]) that was excluded from analyses here. Feeders were designed as enclosed seed trays with two open access points, each fitted with radio-frequency identification (RFID) antennae (Dorset ID) to record the PIT tags of zebra finches as they freely entered and exited the feeders. The only way for the birds to obtain food was to visit these feeders. During a 5-day habituation period to the free-flying aviaries, we checked that all birds regularly visited the feeders and observed no aggressive interactions around the feeder access points. All birds' feeder visits were subsequently logged for 33 days. From this temporal data stream, we extracted bouts of foraging activity using a well-established clustering algorithm [45] to define groups of birds visiting the feeder around the same time. This clustering algorithm generated estimates of flock feeding events lasting on average 290 s (2.5th percentile: 0 s (when birds landed on the feeder entrance and immediately left again) and 97.5th percentile: 610 s). We then calculated association strengths between each dyad of birds in each aviary as the number of observations of both individuals in the same foraging group divided by the number of observations of at least one individual in a foraging group (i.e. the ‘simple ratio index’, ranging from 0 = never observed at the same feeder together to 1 = always observed together; see electronic supplementary methods of [20] for more details) with the asnipe package v. 1.1.3 [46] in r [47]. The social network data can be freely downloaded from Boogert *et al.* [48]. The three social network metrics we extracted as predictors of father–son song similarity were (i) the father–son association strength in each of the 33 daily foraging networks [19]; (ii) the total number and strength of the father's daily associations (i.e. ‘weighted degree’) with all other aviary members excluding the son, as a measure of father ‘gregariousness’ (which could affect his popularity as a song tutor [42]); and (iii) the son's weighted degree excluding the father (as a highly sociable son may be less likely to pay attention to, and thus copy, the father's song). All social network metrics were calculated including both male and female associates, as this reflects their actual social environment and takes into account any influences that female associations may have had on the males' song learning processes. Females were excluded only from the song metrics (see below) as female zebra finches do not sing.

### (c) Song recordings

Male zebra finches each learn one song motif, which is repeated several times to form a song. We recorded the songs of all 17 adult males that were present in the breeding cages when the first chicks started hatching. Only 13 of these males produced fledglings and were present in the aviaries (and thus network analyses), but we also analysed the songs of the unsuccessful breeders (*N* = 4 males), as we could not exclude the possibility that their songs were picked up by fledglings in neighbouring breeding cages. Captive-reared zebra finches tend to learn and produce songs heard between 35 and 65 days post-hatching, but they can incorporate elements heard before or after this sensitive phase [32]. CORT-treated (*N* = 12) and control male (*N* = 8) juveniles’ songs were recorded when juveniles were at least 100 days old (mean ± s.d. = 103 ± 2 days) and their songs had crystallized to become stereotyped (this is known to occur around day 90 post-hatching [49]). Males were induced to sing by presenting each with an unfamiliar female in a sound-attenuated recording room. For additional details, see the electronic supplementary material.

### (d) Song analyses

**Figure 1.**
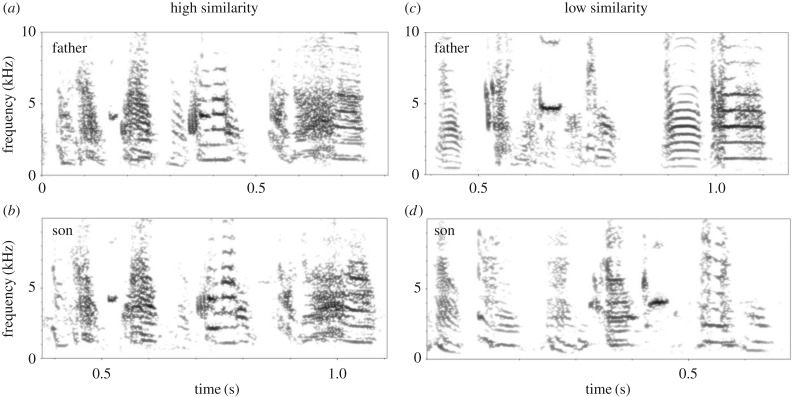
Example songs. Fathers' songs (*a* and *c*) and their sons' songs (*b* and *d*). The spectrograms in (*a*) and (*b*) show a high father–son song similarity (the father's song was the top-ranked model), while (*c*) and (*d*) show a very low song similarity (the father's song was ranked 23rd in similarity to the son's song).

We analysed to what extent the song motif of each juvenile male (recorded once they reached adulthood) matched those of the 19 other juvenile males and of all 17 adult males they were acoustically exposed to. We predicted that most learning would occur from the seven (aviary 1) or six (aviary 2) adult males that fathered the juveniles and/or were present in the same free-flying aviaries from post-hatching day 35 onwards. Song elements were compared using dynamic time warping (dtw) in Luscinia (http://rflachlan.github.io/Luscinia/). This method has previously been applied successfully to zebra finches and other songbird species [23,50,51] to measure broad-scale features of song organization as well as copying accuracy. The resulting dissimilarity matrix between all possible pairs of song elements in the dataset served as the basis for comparisons between individuals' song motifs: for each pair of individuals, we found the best fit between each of one's song motifs and those of the other, and averaged these to generate a motif dissimilarity matrix. For each juvenile, we then ranked all potential song tutors (i.e. other male juveniles and adults, giving ranks 1–36) according to their song dissimilarity scores. We inferred that the male with the lowest dissimilarity score relative to the focal individual's song, and thus the most similar song, was the main song tutor, and this individual was assigned rank 1. The individual with the most dissimilar song (i.e. the largest dissimilarity score) was assigned rank 36. These data can be found in electronic supplementary dataset 1: song similarity scores. Figure 1 shows examples of high and low father–son song motif similarity, and the song analyses are described in more detail in the electronic supplementary material.

### (e) Statistical analyses

To first determine whether there was a link between CORT treatment and juveniles' use of the father as the primary song tutor, we conducted a generalized linear mixed-effects model (GLMM) with binomial error structure. The response variable was whether the juvenile's father was his main song tutor (1) or not (0), the fixed effect was CORT treatment (=1, control treatment = 0) and the random effect was family ID.

Next, to determine whether CORT treatment and father and son's social foraging association metrics during the sensitive phase for song learning correlated with father–son song similarity, we conducted a linear mixed-effects model with a father–son song dissimilarity score as the response variable. Fixed effects were (i) CORT treatment; (ii) the strength of father–son association in each of the daily foraging networks [19]; (iii) the total number and strength of the father's daily associations (i.e. ‘weighted degree’) with all other aviary members excluding the son (to control for ‘father gregariousness'); (iv) the son's weighted degree excluding the father (to control for ‘son gregariousness’); and (v) the number of fledglings in the family (as brood size can affect social learning strategies [12]). We included ‘family ID’ as a random effect, as the 20 juvenile males came from 11 different families (two of the 13 fathers in the aviaries produced only daughters). Previous studies where juvenile males were kept in small song learning groups with a single adult male tutor suggest that the number of male peers present can affect song similarity [52,53]. To test for a correlation between the number of male offspring and father–son song similarity, we used the exact same linear mixed-effects model as described above, but replaced the number of fledglings in the family with the number of male offspring (as these factors were strongly correlated and so could not both be included in the same model). To test whether CORT treatment was related to the strength of father–son associations, we conducted another linear mixed-effects model: the father–son association in the daily foraging networks was the response variable, CORT treatment was the fixed effect and family ID the random effect.

Finally, to test whether CORT treatment was linked to overall song copying accuracy, we conducted a linear mixed-effects model, where the response variable was the song dissimilarity score between juvenile and first-ranked tutor (i.e. with the smallest dissimilarity score), the fixed effect was CORT treatment and the random effect was family ID.

All statistical models were constructed using the ‘lme4’ package v. 1.1–11 in r. To calculate the significance of fixed effects involving network metrics, and account for the fact that individuals' social association metrics are not independent of each other, we used a null models approach [54,55]: we compared the ‘observed’ test statistic, i.e. the coefficient of the slope from the linear mixed-effects model of the observed data, with the distribution of test statistics generated by running the same statistical model on 10 000 permutations of the observed social associations using the r package ‘asnipe’ v. 1.1.3 [46]. These permutations maintain the same data structure as the data collected and only incrementally swap single observations of two individuals occurring in different feeding bouts/flocks [54]. This approach thus maintains, and controls for, aviary ID, the number and ID of individuals in each aviary, the number of times individuals were recorded to visit a feeder and the specific feeder they visited.

## 3. Results

### (a) Link between corticosterone treatment and song tutor choice

We tested whether CORT-treated juveniles were less likely to copy their fathers' song. We found no significant link between CORT treatment and primary song tutor choice (GLMM: slope ± s.e. = −1.077 ± 1.066, *z*_8_ = −1.010, *p* = 0.312). The majority of juveniles (12/20) sang songs most similar to their fathers’ (table 1). Of the eight birds whose songs were most similar to those of alternative tutors, six were CORT-treated juveniles and two were control birds. However, the majority of these eight juveniles' songs were most similar to brothers from the same brood, with the father generally second-ranked (‘father rank: 2’ in table 1) after a brother. The three exceptions in terms of song tutor choice (with father ranked 5th, 15th and 23rd) were all CORT-treated juveniles (table 1). These patterns, illustrated in electronic supplementary material, figure S1, suggest that in some cases, CORT-treated juveniles might avoid their father as a song tutor. Replicating this study with a larger sample size would help establish how robust and biologically meaningful this pattern is.

**Table 1.** Song tutor choice of control and CORT-treated juveniles. The fourth column shows which song similarity ranks the father's song occupied, and the final column shows whom these juveniles copied primarily instead.

juvenile treatment	primary song tutor		father rank	relation to tutor with most similar song
	father	not father^a^		
control	6	*2*	*2, 2*	*brother, unrelated adult*
corticosterone	6	*6*	*2, 2, 2, 5, 15, 23*	*brother, brother, brother, unrelated peer, brother, unrelated adult*

^a^Data in italics refer to juveniles whose songs were not most similar to their fathers'.

### (b) Links between corticosterone treatment, social associations and father–son song similarity

**Figure 2.**
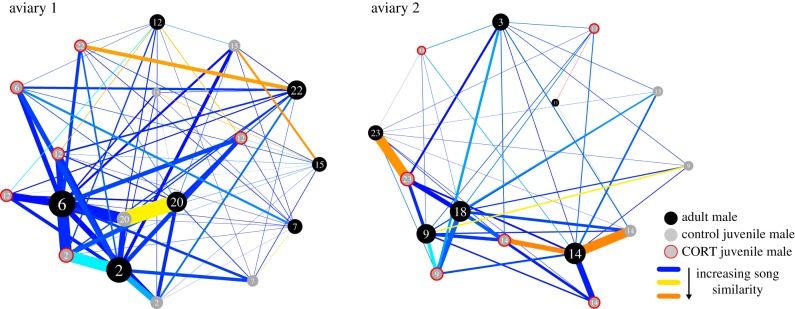
The social foraging associations and song similarities of the males in each of the two aviaries. Our social network metrics and analyses included all males and females in the aviaries, but for the purposes of illustration only males are drawn here, as female zebra finches do not sing. Numbers represent family ID. This figure illustrates that fathers (black circles) and sons (grey circles: controls; with red outline: CORT-treated) with stronger social associations (thicker lines) had more similar songs (redder lines), while more gregarious fathers (larger circles) had sons with less similar songs (bluer lines).

Father–son song similarity was strongly affected by the CORT treatment, with CORT-treated juveniles producing songs that were less similar to their fathers' songs than those of control birds (table 2). Father–son song similarity was also correlated with the strength of father–son social associations (table 2, illustrated for each network day in electronic supplementary material, figure S2): the more often fathers and sons were at the same feeder at the same time (figure 2: thicker lines), the more similar were their songs (figure 2: redder lines). Furthermore, the number and strength of associations between the father and all other aviary members (excluding the son) showed a negative correlation with father–son song similarity: the more gregarious the father (figure 2: larger circles), the less similar his son's song was to his. By contrast, the son's ‘gregariousness’ showed no significant correlation with father–son song similarity. Finally, father–son song similarity was related to brood size: the more fledglings (of both sexes), the more similar the songs of father and son(s). The number of male fledglings in each nest showed no significant relationship with father–son song similarity (table 2). These results are robust to the removal of two outliers, except for the effect of brood size, which was no longer significant (see electronic supplementary material, results and table S1).

**Table 2.** Effect of CORT treatment and social associations on father–son song dissimilarity scores. Full linear mixed-effects model results (*N* = 13 fathers, 20 sons). *P*-values are calculated by comparing the observed slope coefficients with the distribution of slope coefficients from 10 000 permutations of the social network data. Hence, *p-*values do not always exactly match the *t*-statistic (which is a parametric estimate that depends on sample size, which is not defined for social network data). For each fixed effect, the first row of values was generated by the full model, and the second row represents results of the full model but including the number of male offspring instead of the number of fledglings for each zebra finch family. Values in bold indicate significant predictors in both models.

fixed effects	slope	s.e.	95% range of random coefficients	*t*	*p*_rand_
intercept	0.2257 0.1981	0.0223 0.0229		10.115 8.645	
CORT treatment	**0.0234** **0.0234**	**0.0030** **0.0030**	0.0227 to 0.0230 0.0230 to 0.0232	**7.803** **7.777**	**0.016** **0.003**
father–son association	**−0.0346** **−0.0342**	**0.0171** **0.0172**	−0.0308 to 0.0221 −0.0284 to 0.0011	**−2.017** **−1.992**	**<0.001** **0.011**
father gregariousness	**0.0031** **0.0031**	**0.0012** **0.0012**	0.0008 to 0.0030 0.0016 to 0.0027	**2.545** **2.530**	**<0.001** **0.001**
son gregariousness	−0.0020 −0.0020	0.0010 0.0010	−0.0023 to −0.0014 −0.0021 to −0.0016	−1.981 −1.972	0.72 0.292
number of fledglings number of male offspring	−0.0121 −0.0067	0.0061 0.0111	−0.0125 to −0.0122 −0.0069 to −0.0067	−1.973 −0.605	<0.001 0.575
*random effects*	*variance*	*s.d.*		*% total*	
family	0.0007 0.0009	0.0259 0.0305		47.34% 55.50%	

The finding that CORT treatment was associated with reduced father–son song similarity, while father–son association strength increased it (table 2), raises the question whether there might be a direct and negative link between CORT treatment and father–son associations: did CORT juveniles associate less with their fathers as compared to control juveniles? Our post hoc exploration of the data suggested a weak but significant negative relationship between CORT treatment and father–son association strength that emerged when comparing the observed networks with their randomizations (linear mixed-effects model: slope ± s.e. = −0.007 ± 0.009, *t* = −0.758, *p*_rand_ = 0.028). That is, despite being fairly small, the slope parameter of our model was significantly more negative than expected by chance (i.e. although the confidence interval overlaps 0, the slope is outside the 95% range of slopes (−0.0060 to 0.0080) generated by the randomization procedure; see [54] for a detailed explanation of how such patterns can arise). However, absolute father–son association strength differed only slightly between CORT and control juveniles (CORT (*N* = 12): mean ± s.d. = 0.175 ± 0.042; control (*N* = 8): mean ± s.d. = 0.172 ± 0.046). These results should thus be interpreted as CORT-treated juveniles having weaker associations with their fathers *relative to their potential to associate given their social network*.

### (c) Link between corticosterone treatment and overall copying accuracy

When we expanded the analysis to include all primary song tutors, rather than just the father, we found no significant relationship between CORT treatment and overall song learning accuracy: when comparing the songs of juveniles with those of their most similar tutor (i.e. the tutor with the smallest song dissimilarity score; table 1), control and CORT-treated individuals did not differ in their song dissimilarity scores (linear mixed-effects model: slope ± s.e. = 0.007 ± 0.013, *t*_8_ = 0.554, *p* = 0.587). This finding suggests that CORT exposure did not impair juveniles' cognitive ability to learn songs accurately.

## 4. Discussion

The aim of this study was to investigate song learning accuracy and tutor choice of juvenile zebra finch males in free-mixing populations, and the social and hormonal mechanisms that might shape these song learning processes. Our results support the ‘social preference hypothesis’: we found that foraging associations between juveniles and their fathers were strongly correlated with their song similarity. This effect was modulated by early-life stress: young males treated with CORT were slightly less strongly connected to their fathers than expected by chance, and on average, their songs were less similar to those of their fathers when compared to the songs of control males. Our results shed light on the mechanisms by which elevated CORT exposure early in life might have downstream effects on song learning: by modulating social preferences of juveniles and their potential song tutors.

Our results corroborate the results of two previous zebra finch studies showing positive correlations between social associations and tutor–pupil song similarity in an aviary context [42,43], and suggest that the apparent contrasts in tutor choice observed therein may have actually been the by-product of differences in social association patterns. Similar positive correlations between social associations and song or call similarity patterns have been observed in other species, both in captivity (e.g. starlings (*Sturnus vulgaris*) [56]) and in the wild (e.g. song sparrows (*Melospiza melodia*) [57]; Campbell's monkeys (*Cercopithecus campbelli campbelli*) [58]).

We do not claim that our zebra finches were singing (and learning songs) inside the feeders. Instead, our social foraging networks are more likely representative of birds' general social preferences outside the feeders (i.e. by capturing correlations in their behaviour across the day), where singing and song learning presumably occurred. Previous studies suggest that different types of social networks (e.g. proximity versus interaction networks) do not necessarily correlate [59] nor necessarily concur in predicting information transmission [60]. Work is underway to quantify multi-context social networks in zebra finches to assess the domain generality of their foraging associations [61]. In addition, the development of light-weight microphone backpacks [62] offers the exciting possibility of tracking vocal interactions and song development throughout the juveniles’ sensitive phase for song learning in a free-flying context, and thus mapping dynamic social association networks onto dynamic communication networks [63] rather than just the end-product of the crystallized song.

In line with our previous study [19], our results provide greater insights into the effects of early-life CORT exposure on social preferences, in this case reducing father–son foraging associations. Further work tracking individual behaviour in finer detail [61] might be able to reveal the factors and their directionality underlying differences in the potential to associate, such as whether they are driven by the juveniles and/or the fathers. Although included primarily as a control variable, we also found that more gregarious fathers had sons with less similar songs. This could suggest that more gregarious fathers might be less preferred as song models, or perhaps that genetic factors that increase father gregariousness also somehow reduce son song copying accuracy. However, it seems more likely that such gregarious fathers create a more complex social and acoustic environment in which accurate song copying is more challenging for their male offspring. A quarter of the sons were also found to have songs most similar to those of their brothers. This could indicate horizontal social transmission of song, a phenomenon previously described among juvenile peers in small flocks of captive zebra finches containing a single adult song tutor [53]. Alternatively, brothers might not necessarily copy each other's songs directly, but show similar song learning tendencies (e.g. they may, genetically and/or through early-life effects, be predisposed to attend to the same cues in their (social) environment, as seen in mouse sibs [64]), resulting in more similar songs indirectly. It is impossible to distinguish between these hypotheses without further experimental manipulation. Selective feeders, perches or roosting sites (e.g. [65]) could be used to manipulate the gregariousness of fathers as well as father–son and peer bonds, and help to elucidate the potential causal links between social associations/preferences and song learning patterns.

Similar to the pattern we previously observed in the context of socially learning to solve a novel foraging task [20], some CORT-treated sons appear to have sought out song tutors other than the father. This could be because the father may not have been preferred as a role model due to the negative early-life experiences of the CORT-treated offspring in the nest, which would support the ‘tutor choice hypothesis'. Alternatively, fathers may have differentially interacted with CORT-treated and control sons, for example because they perceived their CORT-treated sons to be of lower quality; CORT-treated juveniles weighed less than control juveniles at the end of CORT treatment just before fledging ([19]; electronic supplementary materials) and fathers may have noted this. We hope that recent developments in tracking techniques [61] will help to determine the directionality of this effect (father to son versus son to father) in the future.

Previous studies have suggested that developmental stress may hamper the *ability* of birds to learn their songs accurately [66,67] (although see [68,69]). However, our findings suggest that CORT-induced changes in social preferences, rather than an impaired cognitive ability, could help explain some of the reported tutor–tutee song (dis)similarities. Our results show that CORT-treated juveniles copied their most similar model song as accurately as the control juveniles copied theirs. Similarly, our previous study on the same birds showed that CORT-treated juveniles were faster, not slower, to learn to solve a novel foraging task as compared to the control juveniles [20], as has also been found in another zebra finch population that controlled for foraging motivation through quantifying the metabolic rate [70]. Our findings thus appear to provide no support for the ‘cognitive impairment hypothesis’ in our specific study population (although this could be a false negative (i.e. type II error) due to small sample size) and suggest that stressors may influence song development indirectly as a consequence of their effects on social preferences. Thus, our study has opened up a new window through which to explore the hormonal and behavioural mechanisms underlying information acquisition (i.e. tutor choice) and use (i.e. copying accuracy) in song learning. Unfortunately, our study does not allow us to completely disentangle the intertwined influences of CORT exposure and social preference patterns [19] on song learning due to our limited sample size. In addition, chick sex was unknown at the start of the CORT manipulation, resulting in several broods without control sons. However, our findings provide a useful context to and help to elucidate the contrasting results of previous studies. As a result, we are starting to develop a deeper understanding of factors underlying song learning outcomes.

Stressors experienced early in life clearly affect juveniles' social learning strategies, both when learning about novel food sources [12,20] and when learning about song (this study). Here, by integrating social and communication networks [63], we suggest that changes in social preferences could play a key role in modulating song learning by juveniles; young males that had strong social bonds with their fathers expressed more similar songs. The functional significance and ecological relevance of juveniles (not) copying their fathers’ songs has remained unclear. Most songbirds acquire their songs after dispersing from their natal territory, learning from males other than their fathers, with some species learning during an early critical period, while others continue to learn throughout life [24]. It has been suggested that learning from the father in early development may facilitate later kinship recognition and inbreeding avoidance in wild zebra finches, where extended breeding seasons and high mortality lead to high rates of re-pairing in the colonies [40]. But if a male was successful in producing offspring, why would any of his sons, even if stressed in early development, decide *not* to copy him? Our study suggests that inaccurate/not copying of the father's song may not be a directed strategy by juvenile males, but instead could be a by-product of other social processes. Our findings, when combined with previous studies, clearly highlight the importance of social preference patterns in modulating song learning, and ultimately the links between early-life conditions, social affiliations and information use.

## Supplementary Material

Boogert et al Supplementary methods, results and figures

## Supplementary Material

Boogert et al Song Similarity Scores

## Supplementary Material

Boogert et al Song and Network Data Summary

## Data accessibility

The social network data as first reported in [19] are publicly available via http://dx.doi.org/10.5061/dryad.7qs06. The complete song similarity data and a song-network data summary file have been uploaded as electronic supplementary datasets 1 and 2, respectively, and are published with this manuscript.
