# Supplementary material for: Stress hormones, social associations and song learning in zebra finches
Source: Philos Trans R Soc Lond B Biol Sci. 2018 Aug 13;373(1756):20170290. doi: 10.1098/rstb.2017.0290 (PMC6107560; doi:10.1098/rstb.2017.0290)
Supplement: Boogert et al Supplementary methods, results and figures [file rstb20170290supp1.docx]

METHODS

*Breeding protocol and hormone treatment*

As described in [1], we housed 24 domesticated adult zebra finch pairs from the breeding stock of the University of St. Andrews in plastic-covered steel bar cages (50x50x50 cm) containing a nest box, two perches, a cuttlefish bone, crushed oyster shells, a seed hopper containing mixed finch seed and a water hopper, as well as an open food bowl and an open water bowl. Water was supplemented with Johnsons Vitamin Drops for cage birds. We also provided each pair daily with a tablespoon of Haith’s Egg Biscuit Food and at least once a week with fresh spinach. Birds were maintained at 20±1°C ambient temperature on a 14:10 h light:dark cycle. Opaque partitions prevented visual interactions between pairs in adjacent cages, but all breeding pairs could hear each other and, except for immediate neighbours, also see each other. Of these 24 pairs, 13 produced fertile eggs. To facilitate chick age-standardized hormone treatment we synchronised the within-brood hatching dates of these eggs by replacing them with plastic dummies until the brood was complete. We kept the eggs on cotton wool in the same room, and returned them two days after a female laid her final egg. Upon hatching, chicks were individually marked with nail polish on the head and toe nails. Half of the chicks in each brood were assigned to the corticosterone (CORT) treatment following [2]: between days 12 and 28 post-hatching, they were pipette-fed 20μl of CORT (Sigma Aldrich; 0.155 mg/ml in peanut oil) twice daily, giving a total dose of 6.2 µg CORT/day. This dose is known to result in plasma CORT levels comparable to those naturally induced in untreated zebra finch chicks exposed to an acute stressor [2]. Control chicks were fed 20μl of pure peanut oil when their siblings received CORT. In nests with an uneven number of chicks, the ‘extra’ chick was assigned to the CORT treatment. The experimenter (NJB) treated each chick by briefly removing it from its nest and holding it while pipetting the appropriate liquid into the chick’s beak, after which it was immediately returned to its nest. The temporary nest disturbance caused by this experimental manipulation is therefore similar to that caused by the common practise of weighing chicks to record their growth.

*Song recordings*

All recordings were made in a separate, sound-attenuated room with no visual or acoustic contact to any other zebra finches. We placed each male in a cage (60x44x39 cm high) on one side of a wire-mesh partition (to prevent copulations) while an unfamiliar female was positioned at the other side. Each side of the cage contained a single perch, and we directed the microphone to the centre of the perch where the male tended to sing from. Most males readily sang in the presence of a female, and any male not singing to a particular female was recorded on a subsequent day while being presented with a different female. Changing the female can change a male’s motivation to start singing, but does not affect his song structure, as zebra finches are closed-ended learners whose song structure does not change once it has become crystallized [3]. We made all recordings with a Sennheiser ME66/K6 microphone connected to a Marantz PMD660 recorder. Each song was recorded as an uncompressed .wav file at 44 kHz. Male zebra finches each learn one song motif (in our data-set, consisting of a mean of 13.5 elements, and lasting a mean of 0.638ms), which is repeated several times to form a song. Motifs typically vary little from one rendition to the next, but in some birds there is more substantial variation, typically due to the omission of elements. We therefore selected 4-6 (median 5) motifs per individual for further analysis.

*Song analyses*

A) Comparison of song elements

Song elements were compared using dynamic time warping (dtw) in Luscinia (http://rflachlan.github.io/Luscinia/). This method has previously been applied to zebra finches and other songbird species [4–6] and has successfully measured broad-scale features of song organisation as well as copying accuracy. Each song element was measured from a spectrogram (time step = 0.5ms; 512 spectrogram points; maximum frequency = 10kHz). At each time step, 6 acoustic parameters were measured (see below), and each element was characterised by the multidimensional trajectory of these parameters. To compare elements, these trajectories first needed to be aligned. The dtw algorithm uses a simple dynamic programming technique to search for an optimal alignment between two elements, allowing for stretching or compression of the signal in time. Then an overall dissimilarity between two elements was calculated as the mean of the Euclidean distances between points along the alignment.

The acoustic features used in the analysis were: time, mean frequency, fundamental frequency, normalised fundamental frequency, fundamental frequency change, and Wiener entropy. These features capture different aspects of how elements differ from one another. “Time” captures variation in how elements may be stretched or compressed in time. “Mean frequency” and “Wiener entropy” both capture aspects of spectral complexity. The remaining “Fundamental frequency” features relate to aspects of pitch – both absolute, or in the case of the “normalised” and “change” features, relative pitch.

Other settings of the Luscinia analysis that we used were: compression factor: 0.001; minimum element length: 10. Together these ensured that each element was characterised by 10 data points across its length. Element features were weighted according to the empirical standard deviations of the entire sample of song motifs from all males, except for “Time”, which was weighted according to the longer of the two elements being compared. This ensured that time differences were compared in a manner consistent with Weber’s Law [6]. An additional weighting of 10 was applied to “Time”, since this was required to make it approximately equal in importance to other features (see [6] for more details). A maximum dynamic time warp of 100% was allowed; frequency parameters were log-transformed (again to correspond to Weber’s Law); elements were weighted by their relative amplitude (so that measurements in louder parts of the element played a more important role in the overall dissimilarity score); dynamic warping and interpolation in time warping were selected, and five time alignment points were used (see http://rflachlan.github.io/Luscinia/ for more details). These analyses generated a dissimilarity matrix between all possible pairs of elements in the dataset, which served as the basis for comparisons between individuals’ song motifs.

B) Comparison of individuals’ song motifs

From the element dissimilarity matrix, we calculated dissimilarities between pairs of individuals’ song motifs. To do this, we followed the following four steps: (1) Element dissimilarities were log-transformed. Since our study examined song learning, our interest was mainly in the most similar percentiles of the element dissimilarity matrix, rather than differences between broad element classes. (2) We calculated element transition dissimilarities, defined as: $1/2\left( {d\left( a,b \right)}_{i,j}+{d\left( a,b \right)}_{i+1,j+1} \right)$, where ${d\left( a,b \right)}_{i,j}$ is the dissimilarity between element *i* in motif *a* and element *j* in motif *b*. A low element transition dissimilarity thus reflects that two elements in a row were similar between the two motifs. By using element transitions, we thus captured information about the learning of element sequences as well as element structure. Since zebra finch motifs are typically immediately repeated within a song bout, if the first of the adjacent elements was the final element in the motif, the second of the elements was set to be the first element in the next motif. (3) For each element transition in motif *a*, we then found the most similar element transition in motif *b*. We averaged these across all transitions in the first motif to generate an overall score of motif dissimilarity between motifs *a* and *b*. Since this measure was not symmetric, we re-calculated the song dissimilarity with the identity of the motifs reversed, and then selected the score with the highest overall dissimilarity. The result was a dissimilarity matrix between each pair of song motifs recorded. (4) Finally, for each pair of individuals, we found the best fit between each of one’s motifs and those of the other, and averaged these to generate a dissimilarity matrix between each pair of individuals in the sample.

From the individual-level dissimilarity matrix, we selected the comparisons of interest: the motif dissimilarities between each juvenile and all other males. For each juvenile we then ranked all potential song tutors (i.e. other male juveniles and adults) and inferred that the male with the smallest dissimilarity score to the focal individual’s song was the main song tutor. These data can be found in Supplementary Dataset 1: Song Similarity Scores. Figure 1 shows examples of high and low father-son song motif similarity.

RESULTS

*Links between corticosterone treatment, social associations and father-son song similarity*

Our results could have been influenced by two outliers (defined as values outside 1.5 times the inter-quartile range): two juveniles had father-son song dissimilarity scores of 0.290 and 0.284, respectively, while the inter-quartile range of song dissimilarity scores was 0.171 – 0.199 (with median: 0.182 and mean: 0.194), suggesting they did not copy their father’s song at all. We therefore re-ran the same model but excluding the song data for these two sons (while keeping them in all social network-related calculations where they did not represent outliers). Excluding these two outliers generated qualitatively similar results explaining father-son song dissimilarity, except for the effect of number of fledglings, which was no longer significant (Table S1).

**Table S1.** **Effect of corticosterone (CORT) treatment and social associations on father-son song dissimilarity scores.** Linear mixed-effects model results (N=13 fathers, 18 sons). P values are calculated by comparing the observed slope coefficients with the distribution of slope coefficients from 10,000 permutations of the social network data. Hence P values do not always exactly match the t-statistic (which is a parametric estimate that depends on sample size, which is not defined for social network data). For each fixed effect, the values were generated by the full model but excluding two father-son song dissimilarity outliers. Values in bold indicate significant predictors.

| **Fixed effects** | **Slope** | **SE** | ***t*** | ***P*_rand_** |
| --- | --- | --- | --- | --- |
| Intercept | 0.203 | 0.013 | 15.683 |  |
| **CORT treatment** | **0.006** | **0.002** | **2.835** | **0.002** |
| **Father-son association** | **-0.023** | **0.011** | **-1.995** | **0.002** |
| **Father gregariousness** | **0.003** | **0.001** | **2.996** | **0.036** |
| Son gregariousness | -0.002 | 0.001 | -2.750 | 0.74 |
| Number of fledglings | -0.005 | 0.003 | -1.592 | 0.166 |
| **Random effects** | **Variance** | **SD** | **% total** |  |
| Family | 0.000 | 0.012 | 30.71% |  |

FIGURES

**Figure S1.** Song tutor choices of control (0) and corticosterone-treated juveniles (1). The different colours represent different families. Large circles are juvenile males and small circles adult males. Black arrows point to the individuals with the most similar songs (i.e. primary tutors), while grey arrows point to secondary tutors. Arrow length is arbitrary. This figure illustrates that most juveniles later sang songs most similar to those of their fathers, although some juveniles’ songs were most similar to those of their brothers (e.g. brown family) or to the songs of unrelated individuals (e.g. blue corticosterone-treated (1) juveniles).

**Figure S2. The link between father-son song similarity and association strength**. Each panel shows the daily average father-son association strength for sons whose songs were not most similar to their fathers’ (0), and sons whose songs were most similar to those of their fathers (1). Darker colours indicate larger differences between these two categories of juveniles. Red plots indicate days on which sons with songs most similar to their fathers’ had on average stronger father-son associations than sons with dissimilar songs to their fathers’, blue plots indicate days when the reverse was the case, and plots are grey when daily average father-son association strengths were equal for the two categories of juveniles. This figure illustrates that sons who produced songs similar to their fathers’ also tended to have stronger associations with their fathers during the sensitive phase for song learning as compared to sons with dissimilar songs.

REFERENCES

1. Boogert NJ, Farine DR, Spencer KA. 2014 Developmental stress predicts social network position. *Biol. Lett.* **10**, 20140561.

2. Spencer KA, Evans NP, Monaghan P. 2009 Postnatal stress in birds: a novel model of glucocorticoid programming of the hypothalamic-pituitary-adrenal axis. *Endocrinology* **150**, 1931–4. (doi:10.1210/en.2008-1471)

3. Williams H. 2004 Bird song and singing behavior. *Ann. N. Y. Acad. Sci.* **1016**, 1–30.

4. Lachlan RF, Anderson RC, Peters S, Searcy WA, Nowicki S. 2014 Typical versions of learned swamp sparrow song types are more effective signals than are less typical versions. *Proc. R. Soc. B Biol. Sci.* **281**, 20140252. (doi:10.1098/rspb.2014.0252)

5. Lachlan RF, Verhagen L, Peters S, Cate C ten. 2010 Are There Species-Universal Categories in Bird Song Phonology and Syntax? A Comparative Study of Chaffinches (*Fringilla coelebs*), Zebra Finches (*Taenopygia guttata*), and Swamp Sparrows (*Melospiza georgiana*). *J. Comp. Psychol.* **124**, 92–108. (doi:10.1037/a0016996)

6. Lachlan RF, Verzijden MN, Bernard CS, Jonker PP, Koese B, Jaarsma S, Spoor W, Slater PJB, Ten Cate C. 2013 The progressive loss of syntactical structure in bird song along an Island colonization chain. *Curr. Biol.* **23**, 1896–1901. (doi:10.1016/j.cub.2013.07.057)
